# The Influence of Aquaculture and a Natural Environmental Gradient on Shell Landmark Variation of the Mediterranean Mussel (*Mytilus galloprovincialis* Lamarck, 1819) From the Eastern Adriatic Sea

**DOI:** 10.1002/jmor.70043

**Published:** 2025-03-26

**Authors:** Marina Piria, Ivan Špelić, Slađana Nikolić, Rigers Bakiu, Joanna S. Hamilton, Jonathan P. A. Gardner

**Affiliations:** ^1^ Department of Fisheries, Apiculture, Wildlife Management and Special Zoology University of Zagreb Faculty of Agriculture Zagreb Croatia; ^2^ Department of Ecology and Vertebrate Zoology, Faculty of Biology and Environmental Protection University of Łódź Łódź Poland; ^3^ Institute of Marine Biology University of Montenegro Kotor Montenegro; ^4^ Department of Aquaculture and Fisheries, Faculty of Agriculture and Environment Agricultural University of Tirana Tirana Albania; ^5^ Albanian Centre for Environmental Protection and Sustainable Development Tirana Albania; ^6^ School of Biological Sciences Victoria University of Wellington Wellington New Zealand

**Keywords:** Bivalvia, geometric morphometry, landmark analysis, Mediterranean Sea, Mytilidae, wild and farmed populations

## Abstract

Geometric morphometry is effective in distinguishing bivalve species and populations, including the economically and environmentally important Mediterranean mussel, *Mytilus galloprovincialis*. Although widely distributed, shell shape variation in *M. galloprovincialis* along the eastern Adriatic Sea has been infrequently studied. Farming practices and environmental conditions may affect the development of shell shape, as has been reported in the *Mytilus* genus from numerous locations globally. Building on earlier genetic analyses of mussels collected along a natural environmental gradient, this study aimed to identify shell landmark differentiation between wild and cultured populations and among northern, middle, and southern populations of the eastern Adriatic Sea using a geometric morphometric approach. Samples from 12 sites in Croatia, Montenegro, and Albania, including 4 aquaculture farms, were examined for variation in 9 internal shell landmarks. Wild populations exhibited a more extended posterior adductor muscle scar and a more elongated shape compared to farmed populations. Mussels from low salinity environments in the north and south exhibited an elongated shell shape compared to high salinity environments. Southern populations exhibited an extended posterior adductor muscle scar, along with an elongation of the lateral ligament and ventral umbo orientation that caused a concave shape of the ventral shell border compared to northern populations. The differences in environmental conditions in the Adriatic Sea, such as reduced salinity in Boka Kotorska Bay (Montenegro) in the south and Limski Bay (Croatia) in the north, likely play a role in influencing the variability of shell landmarks. These results may be applied to farming practices so that high‐quality spat are collected from source sites with environmental conditions that match the farm site to which the spat are transferred. Overall, these results provide valuable insight into how *M. galloprovincialis* shell landmarks respond to environmental variation at large (hundreds of kilometres) spatial scale.

## Graphical abstract

We used geometric morphometry to identify shell landmark differentiation in the Mediterranean mussel *Mytilus galloprovincialis* across the eastern Adriatic Sea. The results revealed that an elongated shell shape was particularly characteristic of southern populations and those from environments with lower salinity concentrations. This suggests that environmental conditions, especially reduced salinity, play a role in explaining shell shape variation.

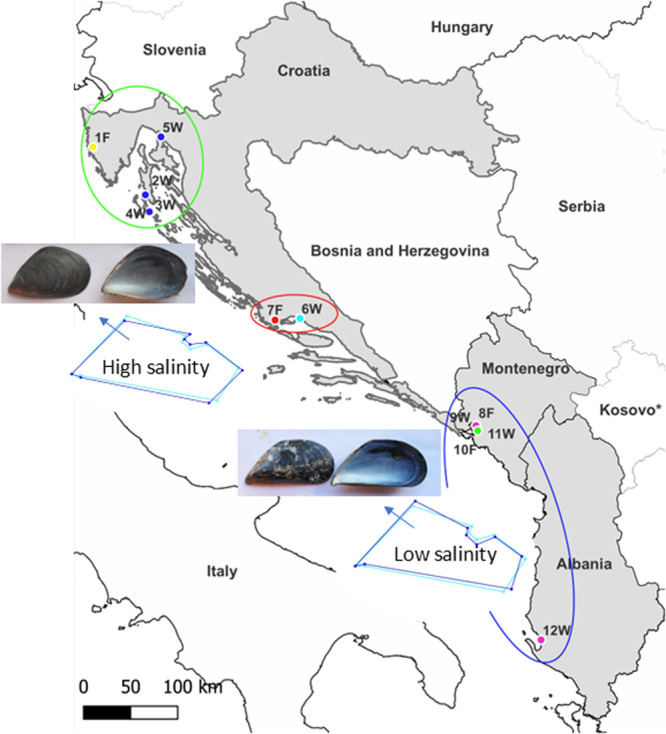

## 1 Introduction

Morphological studies of shell shape variation in bivalve molluscs have been successfully employed for discriminating among populations within a species and between species (Rufino et al. 2006; Morais et al. 2014; Moschino et al. 2015). Over the years, several different approaches to the analysis of shell shape (in a broad sense) have been employed. For example, the earliest descriptions of new species from the 18th and 19th centuries by authorities such as Linnaeus, Lamarck, Gould, Hupé and d'Orbigny relied, in large part, on shell shape differences. Subsequently, a traits‐based approach to species identification and to population assignment was widely employed (e.g., McDonald et al. 1991; Innes and Bates 1999; Gardner 2004), while shell outline (silhouette) methods were also employed (e.g., Ferson et al. 1985; Krapivka et al. 2007; Gardner and Thompson 2009). The most recent approach used in morphological studies of shell shape variation is geometric morphometry, which involves both elliptic Fourier analysis (contour analysis) and landmark‐based methods (e.g., Valladares et al. 2010; Illesca et al. 2018). This is particularly applicable in cases where the diversity of shapes is not large, and the conventional morphometric (trait‐based) approach is less likely to be successful (Van der Molen et al. 2013). While elliptic Fourier analysis has been used reasonably frequently, landmark‐based methods have not been used as often but demonstrate greater power in detecting and quantifying shape differences between closely related species, and between populations of the same species experiencing different environmental conditions (Rufino et al. 2006).

A key strength of geometric morphometric analysis is that it enables the partitioning of shape and size components, thereby preserving the main geometric properties of the specimens. This approach generates a visual representation and determines shape variables that can be analysed statistically (Morais et al. 2014). Bivalves are an excellent taxonomic group for the application of geometric morphometric methods due to their hard shells, which enable manipulation without deformation of shape (Rufino et al. 2006) and which possess many important (e.g., muscle attachment, hinge, ligament) and easily located landmarks (Illesca et al. 2018).

The marine mussel genus *Mytilus* is widely distributed in both the Northern and Southern hemispheres, where it is an ecologically important component of intertidal and subtidal communities and a significant contributor to the aquaculture sector (Michalek et al. 2016; Gardner et al. 2021). In the Northern hemisphere, the genus *Mytilus* includes a complex of three sibling species: the blue mussel *Mytilus edulis* Linnaeus, 1758, a cold‐water and low salinity tolerant mussel *Mytilus trossulus* Gould, 1850, and the Mediterranean mussel *Mytilus galloprovincialis* Lamarck 1819 (McDonald et al. 1991; Gosling 1992; Wilson et al. 2018). *M. edulis* is found in the northeast and northwest Atlantic Ocean basin, whereas *M. trossulus* is circumpolar in the northern Pacific Ocean, northwest Atlantic Ocean and the Baltic Sea (Wonham 2004; Väinölä and Strelkov 2011). The native distribution of *M. galloprovincialis* includes the Mediterranean Sea, and the Atlantic Ocean coasts of northern Africa and western Europe, as far north as SW England and southern Ireland (Gardner and Skibinski 1988; Gardner 1992; Gosling 1992).

In *Mytilus*, analyses of shell shape (i.e., outline) and shell trait (e.g., hinge plate or individual muscle scar size, shape and location on the shell) variation have been used extensively in the examination of both Northern and Southern hemisphere mussels to describe, for example: differences between putative taxa (Lewis and Seed 1969; Seed 1972; Seed 1992; Gardner and Thompson 2009); developmental stability arising from natural inter‐specific hybridisation (Gardner 1995); differences among fossil, midden and contemporary mussels (Gardner 2004); the influence of tidal vertical zonation, tidal currents, and waves, as well as anthropogenic activities on the ecophenotypic variability of shells (Moschino et al. 2015); fluctuating asymmetry at different levels of water pollution (Scalici et al. 2017); and variation along a natural environmental gradient across a pronounced biogeographic boundary (Illesca et al. 2018). Thus, while numerous shape and trait variation studies of *Mytilus* taxa have been conducted, and many of them for *M. galloprovincialis*, the shape variability of *M. galloprovincialis* distributed across the Mediterranean Sea, including the Adriatic Sea, has not been studied much and such analysis has not been applied to the study of shape differentiation of native mussels along the Adriatic Sea latitudinal gradient.

As a native species in the Adriatic Sea, *M. galloprovincialis* represents the only species from the genus *Mytilus* used for aquaculture in the region (Peharda et al. 2007). The largest populations of *M. galloprovincialis* in the eastern Adriatic Sea are found in the Bay of Mali Ston, Boka Kotorska Bay, Krka estuary, Novigrad Sea and Limski Bay (Župan and Šarić 2014; Mandić et al. 2016). The traditional cultivation practice on the eastern Adriatic coast involves collecting spat (juvenile mussels typically 2–5 mm shell length) from the sea on ropes or rafts. During growth and before the final harvest for the market, mussels may be sorted and re‐socked or otherwise manipulated (e.g., to remove fouling organisms) (Robert et al. 2013). This manipulation may interfere with the development of shell shape, as has been observed among *Mytilus chilensis* populations (Valladares et al. 2010). Furthermore, the shape of mussel shells may vary in response to environmental factors due to their plasticity and the possibility of alterations of the whole community in conditions of rapidly changing environments (Scalici et al. 2017; Telesca et al. 2018). In this regard, distribution along a latitudinal gradient may also be an important factor in explaining mussel shell shape variation (e.g., Valladares et al. 2010; Illesca et al. 2018) because ecological conditions vary in the Adriatic Sea on a latitudinal cline from north to south (Lipej and Dulčić 2004; Hamilton et al. 2023). The mean values of annual temperature, salinity and coastal water input in the northern region of the Adriatic Sea differ from the southern region and vary along the coast, from the open shore to the islands (Orlić et al. 1992; Mihanović et al. 2021). Elsewhere, salinity has been identified as the main driver for the spatial effect on *Mytilus* shape, whereas temperature and food supply appear to be important drivers of mussel shape heterogeneity (Telesca et al. 2018). However, despite the widespread anti‐tropical distribution of members of the genus *Mytilus* across the globe and numerous studies of various forms of shape variation, the full effect of environmental variation on shell shape variation remains unknown.

In the present study, our main hypothesis was that regional variation in salinity and coastal water input among the northern, central, and southern sub‐basins of the Adriatic Sea may be an important driver in modulating the morphology of *M. galloprovincialis* shell shape. Using a geometric (landmarks) morphometric approach the current work therefore aimed to identify: (1) the morphometric differentiation between and among wild and farmed populations and (2) the morphometric regional differentiation of *M. galloprovincialis* populations collected from the northern, middle and southern parts of the eastern Adriatic Sea in relation to salinity levels. The results of this study shed light on how environmental variation influences shell shape in *M. galloprovincialis* in its native range and may be important in informing sustainable aquaculture management practices of *M. galloprovincialis* in the Adriatic Sea.

## 2 Materials and Methods

### 2.1 Study Area

The Adriatic Sea is an oligotrophic, semi‐enclosed sea, connected to the Mediterranean Sea by the narrow Strait of Otranto (Ferentinos and Kastanos 1988; Kovačević et al. 1999; Mentaschi et al. 2024). It is divided into the shallow northern basin, extending up to Venice, the middle Adriatic basin, which reaches the Palagruža Sill, and the deepest basin in the south (South Adriatic Pit) extending to the Otranto Strait, where the Ionian Sea begins (Lipej and Dulčić 2004). The Adriatic Sea is situated between the subtropical high‐pressure zone and the mid‐latitude or westerlies belt, with atmospheric disturbances generally moving from west to east. Summer surface water temperatures range from 22°C to 26°C, winter temperatures from ~10°C in the south to ~2°C in the north, with the eastern coast being warmer (by ~2°C) than the western coast (Orlić et al. 1992). The average salinity is 38.3 psu, with the highest measured value being 39.26 psu (Mihanović et al. 2021). Salinity is lower and more variable in the northern region and in coastal zones, and highest in the southern region (Lipej and Dulčić 2004).

The northern Adriatic Sea is shallow and strongly influenced by input from the rivers of northern Italy, particularly the Po (Lipej and Dulčić 2004). The coastal water of the middle Adriatic Sea is mostly influenced by local springs and middle‐sized and small Croatian rivers such as the Zrmanja, Jadro and Žrnovnica. Along the south‐eastern coast the large rivers influencing variation in temperature and salinity of the coastal water are the Neretva in Croatia, the Bojana in Montenegro and the Buna, Drini and Vjosa in Albania (Simeoni et al. 1997; Orlić et al. 2006; Pešić et al. 2019). Boka Kotorska Bay in Montenegro is influenced strongly by sub‐marine freshwater sources and freshwater input from the land, which can strongly modify coastal sea surface temperature and salinity (Bellafiore et al. 2011). The eastern Adriatic Sea is generally oligotrophic, but some coastal areas (mainly bays) may be classified as mesotrophic or eutrophic, and usually such sites are used for aquaculture (Ikica et al. 2017; Đurović et al. 2018).

The mean surface flow of the Adriatic Sea is globally cyclonic, with the Eastern Adriatic Current flowing along the eastern side from the eastern Strait of Otranto to as far north as the Istrian Peninsula (Orlić et al. 1992; Russo and Artegiani 1996). The return flow is the Western Adriatic Current, which flows to the southeast along the Italian coast (Poulain 2001). The global cyclonic circulation is broken into three main re‐circulation cells in the northern, central and southern sub‐basins, with varying seasonal strengths (Artegiani et al. 1997; Poulain 2001; Lipej and Dulčić 2004), influencing the transportation of nutrients and the dispersal of pelagic larvae to the northern, central, and southern sub‐basins (Grilli et al. 2005; Marini et al. 2010; Bray et al. 2017).

### 2.2 Sampling and Sample Preparation

**Figure 1:**
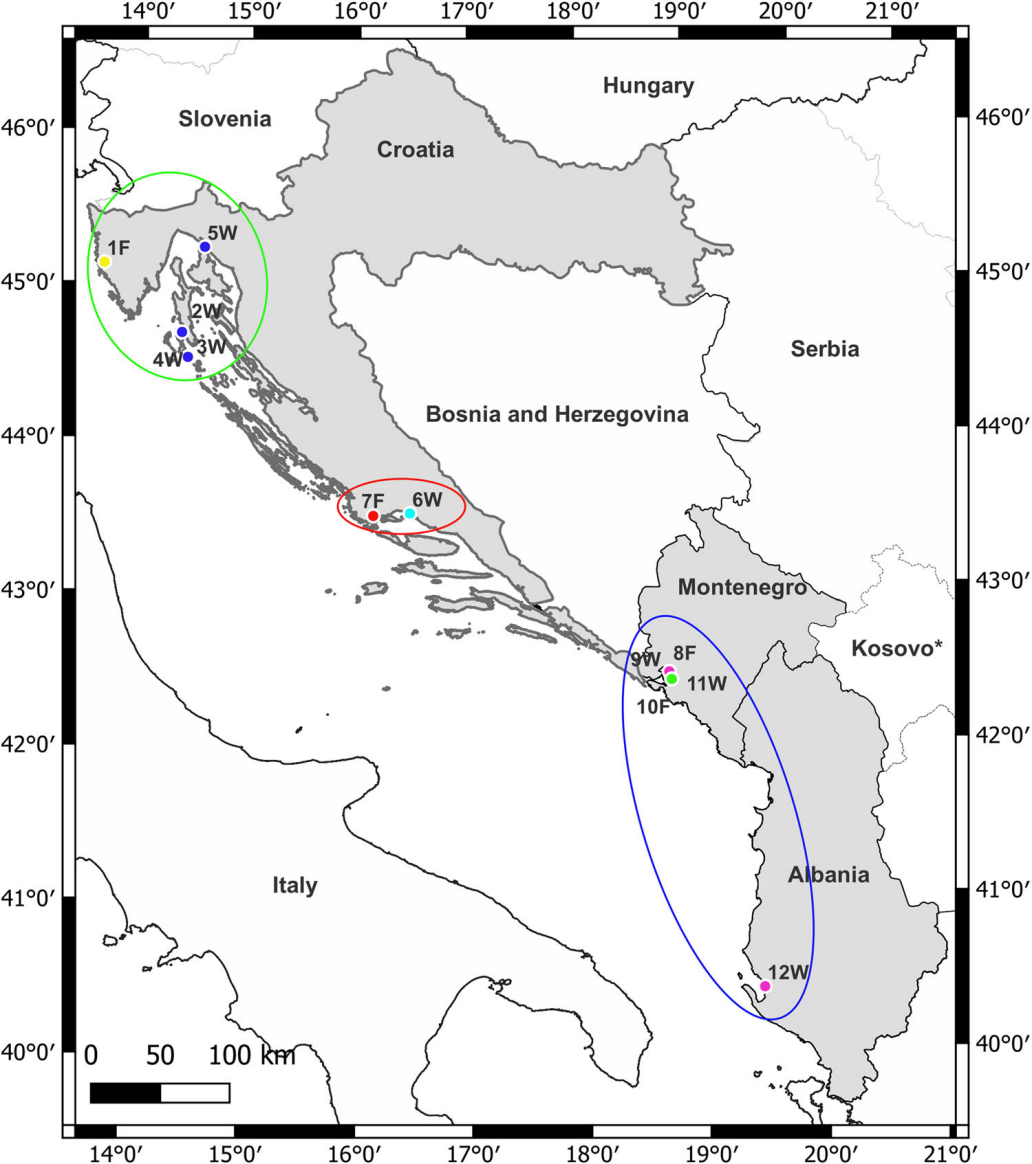
Map of the sampling sites: W = wild; F = farmed; 1 = Limski Bay; 2 = Osor; 3 = Island of Lošinj, Mali Lošinj (Marina; 4 = Island of Lošinj, Mali Lošinj (Čikat); 5 = Črišnjevo; 6 = Split Vranjic Bay; 7 = Split, Poljica Marina; 8 = Boka Kotorska, Orahovac, farm; 9 = Boka Kotorska, Orahovac, near farm; 10 = Boka Kotorska, Dobrota farm; 11 = Boka Kotorska, Dobrota, near farm; 12 = Vlore; the different circle colours denote the north (green), middle (red) and south (blue) populations.

The sampling sites cover the eastern coastline of the Adriatic Sea, including the Istrian Peninsula and Lošinj Island in the north, Kaštela Bay in the middle, and Boka Kotorska and Vlore Bays in the south, spanning three countries: Croatia, Montenegro and Albania. Mussel sampling took place in the late summer and early spring of 2014 and 2015 at the 12 designated sites (Figure 1; Table S1) at depths of up to 1 m. Of these sites, four samples were collected from aquaculture farms, while eight were collected from the wild, found on various substrates such as rocks, concrete piers and floating docks (Table S1). Most of the sampling sites along the Croatian coastline are characterised by high minimum salinities and mild summer temperatures (Kovačić et al. 2017; Hamilton et al. 2023), except for the Istrian Peninsula (Limski Bay), where lower salinity concentrations may occur (Klöppel et al. 2011). The southern sites and lagoons (in Montenegro and Albania), including enclosed bays (such as Limski Bay) in the north, experience high volumes of freshwater input and/or low water exchange with the sea, resulting in reduced salinity concentrations and high summer temperature values (Bellafiore et al. 2011; Kolitari et al. 2013; Đurović et al. 2018; Hamilton et al. 2023).

Following removal of damaged specimens, 35 shells (right valves) per site were used for geometric (landmark) morphometric analysis. Each specimen was cleaned, labelled, and placed on a white background with a visible scale. Photographs of the inner surface of the right valve of each specimen were taken using a digital camera mounted on a camera stand, maintaining a sufficient distance from the shell (300 mm) to reduce the parallax effect (Fruciano 2016).

### 2.3 Geometric Morphometric Analyses

#### 2.3.1 Preparation of Shape Variables

**Figure 2:**
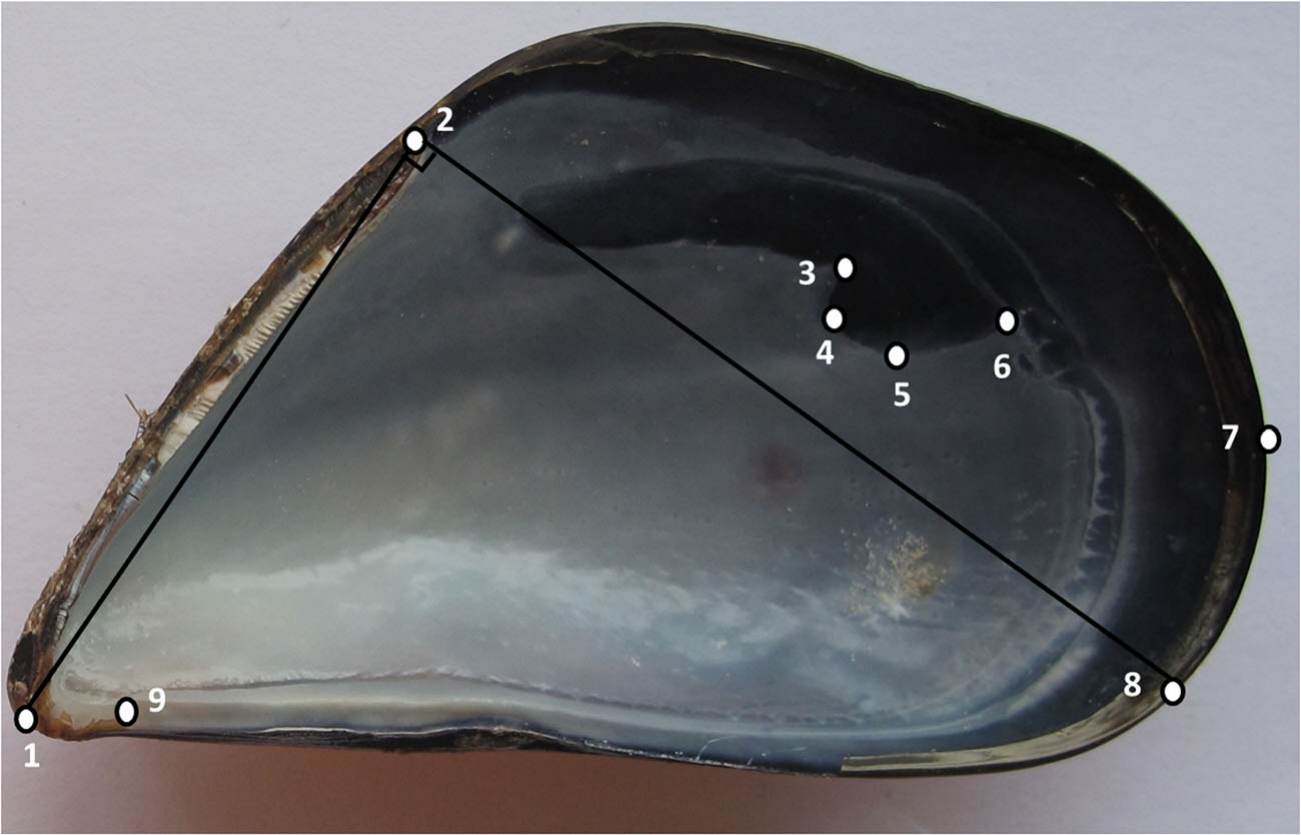
Map of nine homologous landmarks of the right valve of *Mytilus galloprovincialis*: (1) Umbo; (2) Ligament; (3) Posterior Adductor 1; (4) Posterior Adductor 2; (5) Posterior Adductor 3; (6) Posterior Adductor 4; (7) Posterior margin; (8) Maximum distance across shell, from posterior end of ligament scar to ventral margin; and (9) Anterior Adductor (modified from Valladares et al. 2010).

The TpsUtil software was used to build the initial.tps file from the photographs (Rohlf 2015). On each image, nine landmark points located on the shell interior were marked using TPSDig2 version 2.32 software to obtain their x and y coordinates (Rohlf 2017). The selection of landmarks was based on a combination of informative points described by Valladares et al. (2010). In the present context mussel shell ‘shape’ is therefore described by the polygon formed by the nine landmark points (Figure 2).

#### 2.3.2 Removal of Non‐Shape Information

Outliers in the data were explored based on squared Mahalanobis distance values, obtained using MorphoJ software (Klingenberg 2011). Specimens that deviated significantly from the others were excluded before further analysis to avoid biasing the analysis and disproportionately influencing the of mean shapes (Fruciano 2016). The removal of non‐shape information (such as differences due to rotation, translation and scaling) was performed using Generalized Procrustes Analysis (GPA) in MorphoJ software (Klingenberg 2011). The outputs of the GPA procedure are Procrustes coordinates that represent shape variables—two for each landmark, because the photographs are two‐dimensional—and the centroid size that represents the size variable, calculated as the square root of the sum of squared distances of each landmark from their respective centroid (Zelditch et al. 2004). Permutation tests of Procrustes distances and the T‐square statistic were used with Procrustes ANOVA in MorphoJ software (Klingenberg 2011) to test the null hypothesis of equal group means. The Procrustes ANOVA model was specified as Y~X, where Y represents Procrustes‐aligned shape coordinates, and X includes categorical predictors: sampling site, origin (wild and farmed), regions (north, middle and south Adriatic Sea) and salinity level (low, medium, high; described by Hamilton et al. 2023). Significance was assessed using 10,000 permutations. Allometric shape variation in the data was removed in MorphoJ software by regressing shape variables on to centroid size (Figure S1). The resulting residuals produced by the regression were used to generate a covariance matrix and were employed as shape data in subsequent analyses (Klingenberg 2011; Liuti and Dixon 2020).

#### 2.3.3 Shape Analysis and Visualisation

Variation of shell (landmark) shape using shape data residuals was first explored and visualised by principal component analysis (PCA). The possible shape differences between mussels from every site were analysed by canonical variate analysis (CVA) (Bravi et al. 2013). CVA was applied to mussels from all sample sites, where the samples were grouped as farmed and wild, and belonging to the north, middle and south Adriatic Sea regions. Additionally, wild and farmed specimens were grouped and tested separately by CVA to identify the shape differences between the two groups. Discriminant function analysis (DFA) was then applied to the shape data for cross‐validation to assess classification reliability and accuracy (Klingenberg 2011). The CVA scores of wild and farmed populations and the scores of the first two CVA axes of north, middle and south populations were plotted, and the shape changes were visualised by a wireframe graph using the MorphoJ software package (Klingenberg 2011).

## 3 Results

### 3.1 Procrustes Analysis

Fifteen individuals were excluded from analyses because they were identified as outliers. In total, 137 farmed (north 35; middle 34 and south 68) and 268 wild individuals (north 132; middle 35; south 101) of *M. galloprovincialis* were analysed (Table S1). Procrustes ANOVA revealed significant differences in shape between origins (farmed and wild), and among regions (north, middle, south) and also individual sampling sites (Table 1). Significant differences in shape among mussels from sites as a function of salinity concentration were also observed (Table S2). The statistical analyses of shape using Procrustes distances among groups of the CVA and DFA in all combinations were significant (Table S3).

**Table 1:** Procrustes ANOVA results on centroid size and shape effect of mussel individuals by sampling site, region and origin (farmed and wild).

Effect	Sampling site		Region		Origin	
	Centroid size	Shape	Centroid size	Shape	Centroid size	Shape
SS	917.4	0.971	703.0	0.321	111.8	0.341
MS	83.4	0.006	703.0	0.023	55.9	0.012
*df*	11	154	1	13	2	28
F	137.15	21.09	624.95	56.4	21.50	30.14
P (parametric)	< 0.0001	< 0.0001	< 0.0001	< 0.0001	< 0.0001	< 0.0001

Abbreviations: *df*, degrees of freedom; F, F ratio; MS, mean squares; SS, sum of squares.

### 3.2 Shape Variability Between Farmed and Wild Individuals

**Figure 3:**
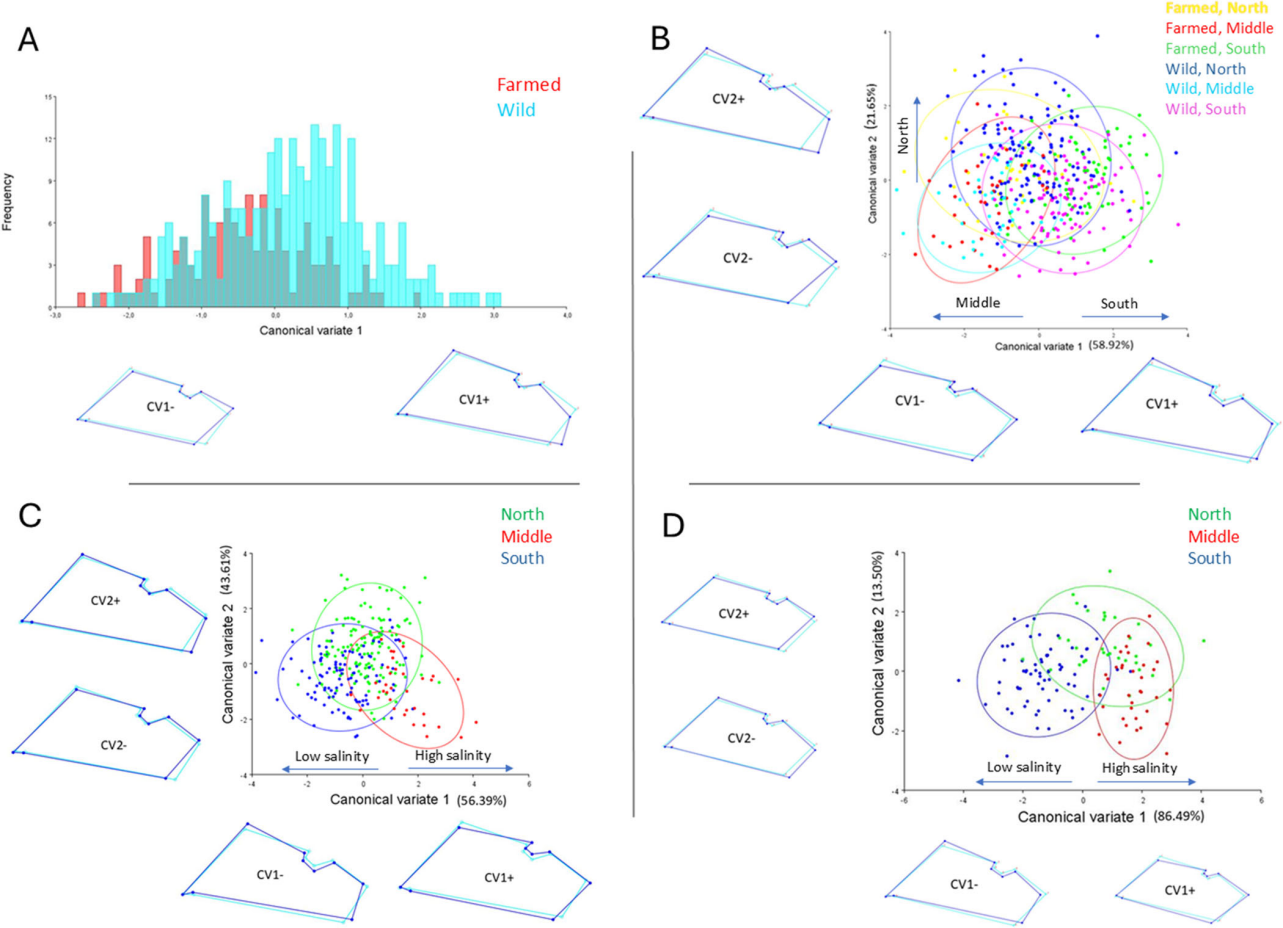
Canonical variate analysis (CVA) depicting shape variation in *Mytilus galloprovincialis* (A) frequency values of the first canonical variate axis between wild and farmed specimens. Scatterplot of the first two canonical variate axes, with equal frequency ellipses classified by region for: (B) farmed and wild; (C) wild; and (D) farmed specimens. Wireframe graphs with nine marked landmarks (see Figure 2) represent shape changes along the first and second CV axes, ranging from negative to positive extremes. The light blue outlines represent the average shape, while the dark blue outlines represent extreme shape changes. For details on farmed and wild population sites and their corresponding codes, refer to Figure 1 and Table S1.

Individuals were classified and subsequently tested as being derived from either farmed or wild populations. The first canonical variate explained variation in shape between wild and farmed populations (Figure 3A). A cross‐validation test of DFA showed 58% and 62% correctly classified specimens for farmed and wild individuals, respectively (Table S3). From the wireframe graph it is apparent that the majority of wild mussels possessed an extended posterior adductor muscle scar (landmarks 3, 4, 5, 6), elongation of the lateral ligament (landmarks 1, 9) and a more elongated shape compared to the farmed mussels. A slight umbo orientation towards the ventral shell margin was characteristic of wild mussels (Figure 3A).

### 3.3 Shape Variability Among Regions and by Origin

Individuals were classified and tested as being derived from north, middle and south populations (farmed and wild). The first two canonical variates (CV1 58.92% and CV2 21.65%) explained 80.57% of the total variation in shape (Figure 3B). A cross‐validation test within the DFA showed that shape differences were least pronounced between farmed and wild specimens of the same region (Table S3). On the wireframe graphical representation of CV1, the southern populations possessed an extension of the posterior adductor muscle scar (landmarks 3, 4, 5, 6), an elongation of the lateral ligament (landmarks 1, 2) and ventral umbo orientation (landmark 1) that caused a concave shape of the ventral shell border (landmarks 1, 9). The southern populations exhibited a more elongated shape compared to the middle region populations. However, northern populations on CV2 exhibited elongation in shell shape but not a visible ventral umbo orientation as was the case for the southern individuals (Figure 3B).

Because there was considerable overlap in shape among wild and farmed individuals from the same region, the shape variations among populations were analysed separately for wild and farmed individuals. The CVA explained 99.99% (CV1 56.39% and CV2 43.60%) variability on the first two axes for wild populations, and 99.99% (CV1 86.49% and CV2 13.50%) for farmed populations classified by region, respectively. Almost all wild populations exhibited an extended posterior adductor muscle scar. The umbo orientation towards the ventral shell margin was associated with southern populations and sites with low salinity concentration on CV1 (Dobrota and Vlore). CV2 exhibited an elongated shape in some individuals from the north (Figure 3C). The middle and southern farmed populations were well separated in the analysis. Southern populations (Dobrota and Orahovac) exhibited a pronounced concave ventral shell border, elongated shape and extended posterior adductor muscle, particularly in comparison to the population from the middle region (Poljica). The majority of northern populations overlapped with the southern populations and showed an elongated shape and extended posterior adductor muscle (Figure 3D).

## 4 Discussion

We identified significant variations in shell shape (as defined by landmark analysis) between wild and farmed *M. galloprovincialis* populations at sites along the eastern Adriatic Sea. The results document that wild populations exhibited an extended posterior adductor muscle scar and a more elongated shell shape compared to farmed populations. In a latitudinal context, elongated shell shape was particularly characteristic of southern populations and those from environments with lower salinity concentrations. This suggests that environmental conditions, especially reduced salinity, play a role in explaining shell shape variation. These findings align with previous studies on mussel shape variation, which showed that shell shape variability along a latitudinal gradient is influenced by environmental conditions (Krapivka et al. 2007; Valladares et al. 2010; Illesca et al. 2018; Telesca et al. 2018).

### 4.1 Shell Shape Variation Between Farmed and Cultured Populations

The extended posterior adductor muscle scar, more elongated shell shape and elongation of the lateral ligament were identified in the majority of *M. galloprovincialis* wild specimens, particularly in the south and in the north. The growth form in the wild environment results in the formation and/or strengthening of body structures such as larger adductor muscles and thicker shells, which are related to increased individual fitness that may be associated with higher predation pressure (Reimer and Harms‐Ringdahl 2001; Kirk et al. 2007; Valladares et al. 2010). This may indicate that energy can be preferentially allocated towards greater muscle growth, which is directly related to individual survival (Valladares et al. 2010). The farmed populations of this study that grow in conditions with high freshwater inputs and low salinity concentrations showed a pattern similar to the majority of wild individuals. The farm sites are mainly located along the mainland coastline in sheltered bays. The primary impediment to mussel farming at more open sites (e.g., offshore islands) is the oligotrophic state of the Adriatic Sea, as well as conditions of higher salinity than are required for maximal mussel growth (Šarušić 2000). For example, the average salinity in the open sea (e.g., at offshore island sites) is around 38.5 psu but the optimum salinity range for mussel farming require 25–28 psu (Župan and Šarić 2014), which is only available along the coastline (i.e., closer to mainland sheltered bays and lagoons). Thus, the siting of farms in more sheltered and more productive nearshore locations will contribute to the observed variation in shell shape between farmed and wild mussels. Furthermore, the shallow‐water farmed mussels exhibit dorsoventral compression, which progressively declines with increasing depth (Michalek et al. 2021). Salinity of the marine environment increases with depth, which was also observed by Klöppel et al. (2011) in Limski Bay. Indeed, all samples in our study were collected from the surface layer to obtain comparable samples. The mussels sampled at farming sites with moderate salinity (Poljica farm) do not express an elongated shape which confirms previous findings.

Limited variation in shell shape among wild and farmed individuals from the same region, particularly for the northern and southern regions, was observed in this study. Such results may reflect traditional aquaculture practice, which includes spat transfer from natural spawning grounds to aquaculture sites with favourable conditions for growth (Kovačić et al. 2017). The farming of mussels in Boka Kotorska Bay and Limski Bay relies exclusively on the collection of spat from nearby sites (Mandić et al. 2016), which may be one explanation of the high overlap in shell shape between wild and farmed samples. However, a limitation of this study was the unequal number of wild and farmed populations in the northern and middle Adriatic Sea regions and the sampling of wild specimens too close to farm populations, such was in Boka Kotorska Bay, which may have resulted in low shape diversity due to similar ecological conditions.

As noted earlier, the farmed mussels in the Adriatic Sea are collected as spat from wild (natural) stocks and are transferred to farm sites, and as a consequence, recent population genetic analyses have revealed that there are only limited genetic differences among *M. galloprovincialis* populations at a spatial scale from northern Croatia to central Albania (Hamilton et al. 2023). Thus, the differences in shape between wild and farmed mussels reported here are unlikely to be genotypic in nature but are most likely to result from two separate and inter‐related effects. First, the effect of the different culturing activities (e.g., rope culture, stocking density, cleaning and reseeding) will result in shape differences for farmed mussels when compared to wild (e.g., rocky reef, dock or pontoon) mussels. Second, the diverse ecological conditions between the coastal (better suited to farming) and open sea conditions (less suited to farming) of sites along the eastern coast of the Adriatic Sea (Kovačić et al. 2017; Đurović et al. 2018) will also have contributed to the shape variation.

### 4.2 Latitudinal Variation in Shell Shape

Mussels from southern Adriatic Sea populations (particularly Dobrota and Orahovac), both farmed and wild, exhibited concave ventral shell borders, an elongated shape and extended posterior adductor muscle scars. Similar results were observed in Chilean and Atlantic *Mytilus* populations (Krapivka et al. 2007; Telesca et al. 2018). In Chile, *M. chilensis* shell shape differences were found in the origin of samples (expansion of the posterior adductor muscle scar, elongation of the lateral ligament and ventral umbo position in noncultivated samples) and along a latitude cline (more elongated shells and more extended posterior adductor muscle scar in most southern samples when compared to the northernmost ones), (Valladares et al. 2010). Subsequently, Illesca et al. (2018) also identified latitudinal differences in the shell shape of *M. chilensis*, with distinct northern and southern forms found in the region of a known biogeographic break between cooler southern and warmer northern waters. Their study shows that specimens from higher (southern) latitudes exhibit a more elongated shell shape and a more pronounced dorsal region compared to those from northern latitudes. Similarly, Telesca et al. (2018) detected shape responses to less favourable environmental conditions at different scales of analysis in Atlantic *Mytilus* populations, indicating the formation of elongated and narrow shells, with more parallel dorsoventral margins. Overall, this pattern of shell shape variation for different *Mytilus* species from different regions is likely to be associated with a latitudinal salinity cline, where environmental conditions with lower salinities produce shells that are more elongated, narrower and with more parallel dorsoventral margins (Krapivka et al. 2007; Telesca et al. 2018).

### 4.3 Influence of Salinity on Shape Variation

Samples from the southern region in our study were collected in Boka Kotorska Bay, which is well known for salinity and temperature variations due to the strong influence of underground freshwater sources (Bellafiore et al. 2011). Similar to the salinity‐related differences in shell shape described by Telesca et al. (2018), we found that a large number of individuals of northern populations (lower salinity values) exhibited an elongated shape. Indeed, salinity concentrations are variable in the northern part of the Adriatic Sea (Lipej and Dulčić 2004), particularly in Limski Bay where the input of the underground freshwater forms brackish water with a high concentration of oxygen and reduced salinity (Klöppel et al. 2011). Similar latitudinal variation has been observed in the shell‐shape of *Brachidontes* mussels from Argentina (Aguirre et al. 2006), where it was suggested that the pattern was related to a decline in salinity towards the north due to the influence of the Rio de La Plata and other smaller rivers (Aguirre et al. 2006). This implies that mussel growth (possibly across different genera) in low salinity conditions results in a more elongated shape and that this variability is not dependent on an environmental gradient per se, but on the freshwater inputs at any given site.

### 4.4 Implications for Farming Practices

Our results may be applied to farming practices that require the identification of spat from collecting sites that are environmentally similar to the farm sites where the spat will be transferred and on‐grown. Seascape genetics analysis for *M. galloprovincialis* in the eastern Adriatic Sea revealed a significant gradient in allelic and genotypic frequencies at a specific microsatellite locus closely tied to environmental variations along the coastline, especially in regions with low salinity concentrations (Hamilton et al. 2023). The results reported by Hamilton et al. (2023) suggest that juvenile mussels collected from areas with low environmental variability have a lower proportion of the favourable MGE7^243^ allele compared to juveniles collected from areas with high environmental variability. Consequently, such mussels are more likely to experience selective mortality. By linking our shell shape analysis results to the seascape genetics analysis results (Hamilton et al. 2023), we can predict that the presence of elongated shells in a spat‐collecting area may indicate appropriate genetic material for aquaculture purposes, given that farms are situated in areas with lower salinity water levels. Such practice may help in finding suitable genetic material and likely reduce natural mortalities in farming facilities.

## Author Contributions

**Marina Piria:** conceptualization, data curation, resources, formal analysis, ivestigation, methodology, writing – review and editing, writing – original draft, funding acquisition, validation. **Ivan Špelić:** methodology, software, writing – review and editing, validation. **Slađana Nikolić:** data curation, ivestigation, writing – review and editing. **Rigers Bakiu:** data curation, investigation, writing – review and editing. **Joanna S. Hamilton:** data curation, writing – review and editing. **Jonathan P. A. Gardner:** data curation, writing – review and editing, conceptualization, funding acquisition, resources, supervision, methodology, investigation, formal analysis, validation.

## Conflicts of Interest

The authors declare no conflicts of interest.

### Peer Review

The peer review history for this article is available at https://www.webofscience.com/api/gateway/wos/peer-review/10.1002/jmor.70043.

## Supporting information

Supporting information.

## Data Availability

The data that support the findings of this study are available on request from the corresponding author. The data are not publicly available due to privacy or ethical restrictions.
