# Supplementary material for: The Influence of Aquaculture and a Natural Environmental Gradient on Shell Landmark Variation of the Mediterranean Mussel (Mytilus galloprovincialis Lamarck, 1819) From the Eastern Adriatic Sea
Source: J Morphol. 2025 Mar 26;286(4):e70043. doi: 10.1002/jmor.70043 (PMC11947508; doi:10.1002/jmor.70043)
Supplement: Supplementary file 1 — Supporting information. [file JMOR-286-e70043-s001.docx]

**SUPPORTING INFORMATION**

Table S1: Sampling sites, regional classification of the sites, sampling date, number of specimens (n), shell length in cm (min=minimal, max=maximal), coordinates (latitude and longitude), origin (farmed or wild), substrate type and yearly fluctuation of salinity at sites from which *Mytilus galloprovincialis* were collected from the eastern Adriatic Sea.

| Country | Sampling site (Abbreviation in Figure 1) | Regional classification | Sampling date | n | Shell length (cm) | | Coordinates | | Origin | Substrate | Salinity  (Hamilton et al., 2023) |
| --- | --- | --- | --- | --- | --- | --- | --- | --- | --- | --- | --- |
|  |  |  |  |  | min | max | Latitude, N | Longitude, E |  |  |  |
| Croatia | Istria Limski Bay farm (1F) | North | 27 Oct 2014 | 35 | 4.5 | 8.5 | 48.133414 | 13.666667 | Farmed | Longline ropes | Low |
|  | Osor (2W) | North | 09 Sep 2015 | 35 | 0.8 | 6.3 | 44.692544 | 14.392286 | Wild | Rocks | High |
|  | Island of Lošinj, Mali Lošinj, marina (3W) | North | 09 Sep 2015 | 35 | 1.1 | 3.4 | 44.532361 | 14.468406 | Wild | Concrete pier | High |
|  | Island of Lošinj, Mali Lošinj, Čikat (4W) | North | 09 Sep 2015 | 35 | 1.5 | 6.0 | 44.531800 | 14.450953 | Wild | Floating dock | High |
|  | Črišnjevo (5W) | North | 10 Sep 2015 | 35 | 1.0 | 4.3 | 45.249400 | 14.582528 | Wild | Rocks | Moderate |
|  | Split Vranjic bay (6W) | Middle | 29 Oct 2014 | 35 | 2.6 | 5.1 | 43.531797 | 16.466539 | Wild | Concrete pier | Moderate |
|  | Split mussel farm, Poljica marina (7F) | Middle | 29 Oct 2014 | 35 | 5.4 | 7.3 | 43.515425 | 16.139222 | Farmed | Longline ropes | Moderate |
| Montenegro | Boka Kotorska, Orahovac, farm (8F) | South | 14 Sep 2015 | 35 | 4.4 | 7.2 | 42.485389 | 18.745961 | Farmed | Longline ropes | Low |
|  | Boka Kotorska, Orahovac, near farm (9W) | South | 14 Sep 2015 | 35 | 3.5 | 5.9 | 42.485678 | 18.743486 | Wild | Concrete pier | Low |
|  | Boka Kotorska, Dobrota, farm (10F) | South | 15 Sep 2015 | 35 | 3.8 | 7.0 | 42.437047 | 18.763208 | Farmed | Longline ropes | Low |
|  | Boka Kotorska, Dobrota, near farm (11W) | South | 15 Sep 2015 | 35 | 2.3 | 3.6 | 42.435958 | 18.763672 | Wild | Concrete pier | Low |
| Albania | Vlore (12W) | South | 15 Sep 2015 | 35 | 1.7 | 3.5 | 40.421711 | 19.488028 | Wild | Rocks | Low |

Table S2. Procrustes ANOVA results on centroid size and shape effect of the individuals by the salinity classifier.

| Effect | Centroid size | Shape |
| --- | --- | --- |
| SS | 147.5 | 0.451 |
| MS | 73.8 | 0.016 |
| df | 2 | 28 |
| F | 29.38 | 38.92 |
| P (parametric) | <0.0001 | <0.0001 |

(SS – sum of squares, MS – mean squares, df – degrees of freedom, F – F ratio)

Table S3. Results of Canonical Variate Analysis (CVA) and Discriminant Function Analysis (DFA) statistic tests for groups of *Mytilus galloprovincialis* populations and results of cross-validation test in DFA for the groups. Numbers in brackets represent the percentage of correctly assigned number of individuals in relation to the total number of compared individuals

| Compared groups | Canonical Variate Analysis (CVA) | | Discriminant Function Analysis (DFA) | | | | DFA cross-validation test |
| --- | --- | --- | --- | --- | --- | --- | --- |
|  | Procrustes distance among group | Permutation test p-value | Procrustes distance | Permutation test p-value | T-square | T-square p-value (permutation test) |  |
| **All samples by origin** |  |  |  |  |  |  |  |
| Farmed-Wild | 0.0202 | 0.0005 | 0.0202 | <0.001 | 36.908 | 0.002 | (58%) 80/137-(62%) 166/268 |
| **All samples by region** |  |  |  |  |  |  |  |
| Farmed, Middle -- Farmed, North | 0.0478 | 0.0002 | 0.0477 | <.0001 | 57.1581 | <0.0001 | (65%) 22/34 – (67%) 24/35 |
| Farmed, Middle -- Farmed, South | 0.0605 | <.0001 | 0.0605 | <.0001 | 263.6674 | <0.0001 | (94%) 32/34 – (88%) 60/68 |
| Farmed, Middle -- Wild, Middle | 0.0302 | 0.0252 | 0.0301 | 0.0160 | 79.5955 | <0.0001 | (79%) 27/34-(69%) 24/35 |
| Farmed, Middle -- Wild, North | 0.0706 | <.0001 | 0.0705 | <.0001 | 94.3365 | <0.0001 | (74%) 25/34-(79%) 104/132 |
| Farmed, Middle -- Wild, South | 0.0581 | <.0001 | 0.0580 | <.0001 | 150.7073 | <0.0001 | (85%) 29/34-(85%) 86/101 |
| Farmed, North -- Farmed, South | 0.0304 | 0.0015 | 0.0303 | <.0001 | 114.5449 | <0.0001 | (80%) 28/35-(81%) 55/68 |
| Farmed, North -- Wild, Middle | 0.0396 | 0.0020 | 0.0395 | 0.0020 | 100.1903 | <0.0001 | (77%) 27/35-(74%) 26/35 |
| Farmed, North -- Wild, North | 0.0278 | 0.0205 | 0.0277 | 0.0230 | 27.5498 | 0.0380 | (51%) 18/35-(70%) 92/132 |
| Farmed, North -- Wild, South | 0.0297 | 0.0026 | 0.0296 | 0.0050 | 79.3129 | <0.0001 | (71%) 25/35-(82%) 83/101 |
| Farmed, South -- Wild, Middle | 0.0516 | <.0001 | 0.0515 | <.0001 | 264.1705 | <0.0001 | (91%) 62/68-(94%) 33/35 |
| Farmed, South -- Wild, North | 0.0290 | 0.0002 | 0.0289 | <.0001 | 121.1187 | <0.0001 | (76%) 52/68-(77%) 102/132 |
| Farmed, South -- Wild, South | 0.0103 | 0.3548 | 0.0102 | 0.3220 | 61.1973 | <0.0001 | (68%) 46/68-(65%) 66/101 |
| Wild, Middle -- Wild, North | 0.0576 | <.0001 | 0.0575 | <.0001 | 109.5208 | <0.0001 | (77%) 27/35-(80%) 105-132 |
| Wild, Middle -- Wild, South | 0.0481 | <.0001 | 0.0480 | <.0001 | 137.1101 | <0.0001 | (89%) 31/35-(83%) 84/101 |
| Wild, North -- Wild, South | 0.0273 | 0.0002 | 0.0273 | <.0001 | 112.4877 | <0.0001 | (69%) 91/132-(72%) 73/101 |
| **Wild by region** |  |  |  |  |  |  |  |
| North-Middle | 0.0664 | <0.0001 | 0.0664 | <0.001 | 119.4138 | <0.001 | (83%) 112/135  (74%) 26/35 |
| South-Middle | 0.0505 | <0.0001 | 0.0505 | <0.001 | 136.2199 | <0.001 | (79%) 82/104  (88%) 31/35 |
| South-North | 0.0308 | <0.0001 | 0.0307 | <0.001 | 113.5965 | <0.001 | (70%) 76/104  (67%) 91/135 |
| **Farmed by region** |  |  |  |  |  |  |  |
| Middle-North | 0.0532 | <0.0001 | 0.053 | <0.0001 | 52.6037 | 0.0016 | (68%) 24/35  (60%) 21/35 |
| Middle - South | 0.0694 | <0.0001 | 0.069 | <0.0001 | 270.810 | <0.0001 | (94%) 33/35  (88%) 61/69 |
| North - South | 0.0325 | <0.0001 | 0.032 | <0.0001 | 112.876 | <0.0001 | (83%) 29/35  (83%) 57/69 |


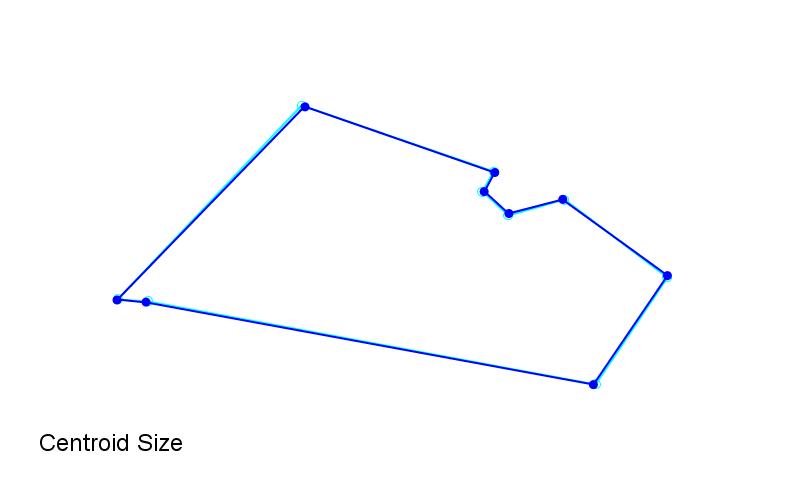


Figure S1. Removed shape change associated with centroid size (allometric shape variation). Light blue outline represents average shape and dark blue represents shape change associated with larger centroid size (larger individuals). A total of 10,000 random permutations were conducted and the multivariate regression result was highly significant (p<0.0001).
